# Supplementary material for: DFT and TD-DFT Investigations for the Limitations of Lengthening the Polyene Bridge between N,N-dimethylanilino Donor and Dicyanovinyl Acceptor Molecules as a D-π-A Dye-Sensitized Solar Cell
Source: Int J Mol Sci. 2024 May 21;25(11):5586. doi: 10.3390/ijms25115586 (PMC11172313; doi:10.3390/ijms25115586)
Supplement: Supplementary file 1 [file ijms-25-05586-s001.zip › ijms-3011105-supplementary.pdf]

Table S1 TD-DFT calculation of the maximum absorption wavelength based on the optimized structure of n = 2 dye by different three functional methods.

Method	λ_{max} (nm)
Experimental	527
B3LYP	461
WB97XD	453
B3PW91	441

Table S2. The calculated dihedral angles between the donor, the polyene bridge, and the acceptor for the studied dyes.

Comp	φ_D	φ_0	φ_1	φ_2	φ_3	φ_4	φ_5	φ_6	φ_7	φ_8	φ_9	φ_A
n =1	-180	-179.99	—	—	—	—	—	—	—	—	—	179.99
n=2	179.99	-179.99	-180	—	—	—	—	—	—	—	—	-180
n=3	-180	179.99	180	179.99	—	—	—	—	—	—	—	-180
n=4	179.97	-179.99	179.99	179.99	179.99	—	—	—	—	—	—	-180
n=5	-180	179.99	179.99	179.99	179.99	-180	—	—	—	—	—	-180
n=6	-180	-180	179.99	179.99	180	179.99	179.99	—	—	—	—	179.99
n=7	-180	179.98	179.99	179.99	179.99	179.99	179.99	179.99	—	—	—	-180
n=8	-180	179.99	179.99	180	-180	-180	179.99	179.99	-180	—	—	179.99
n=9	-180	179.99	179.99	179.98	-180	179.99	-180	-180	-180	179.99	—	180
n=10	179.99	179.98	-180	-180	179.99	179.99	179.99	179.99	179.99	-180	180	-180

Table S3. The calculated dihedral angles between the donor, the polyene bridge, and the acceptor for the studied dyes n =11 to n = 13.

**Comp**	Φ_D	ϕ_0	ϕ_1	ϕ_2	ϕ_3	ϕ_4	ϕ_5	ϕ_6	ϕ_7	ϕ_8	ϕ_9	ϕ_{10}	ϕ_{11}	ϕ_{12}	Φ_A
**n =11**	**-179.99**	**179.98**	**179.99**	**179.99**	**179.99**	**179.99**	**179.99**	**-179.99**	**-179.99**	**179.99**	**179.99**	**179.99**	—	—	**-179.99**
**n= 12**	**-179.99**	**179.99**	**179.99**	**-180**	**-180**	**179.99**	**179.99**	**179.99**	**179.99**	**179.99**	**-180**	**179.99**	**-179.99**	—	**-179.99**
**n= 13**	**179.99**	**180.00**	**179.99**	**-180.00**	**-180.00**	**179.99**	**179.99**	**179.99**	**179.99**	**179.99**	**-180.00**	**179.99**	**-179.99**	**-179.99**	**179.99**

.

Table S4. The energies of HOMO and LUMO, band gap (E g), ionization potential (IP), and electron affinity (EA) for dyes n = 11 to n = 13.

Compounds	E_{HOMO} (eV)	E_{LUMO} (eV)	ΔE (eV)	IP (eV)	EA (eV)
n =11	-4.79	-3.31	1.48	4.79	3.31
n = 12	-4.75	-3.33	1.42	4.75	3.33
n =13	-4.72	-3.34	1.38	4.72	3.34

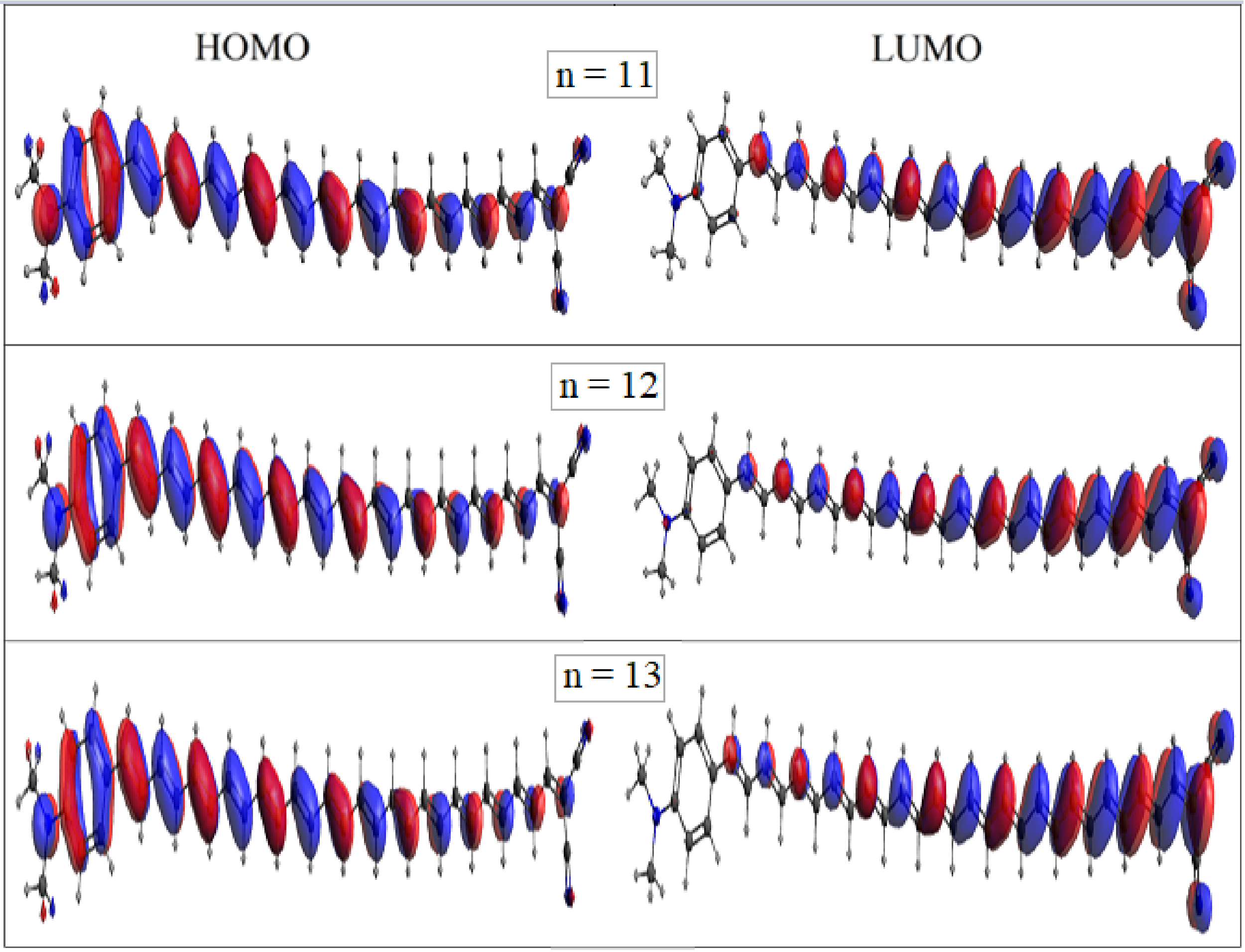

Figure S1. HOMO and LUMO frontier molecular orbital distribution of dyes n =11 to n =13.

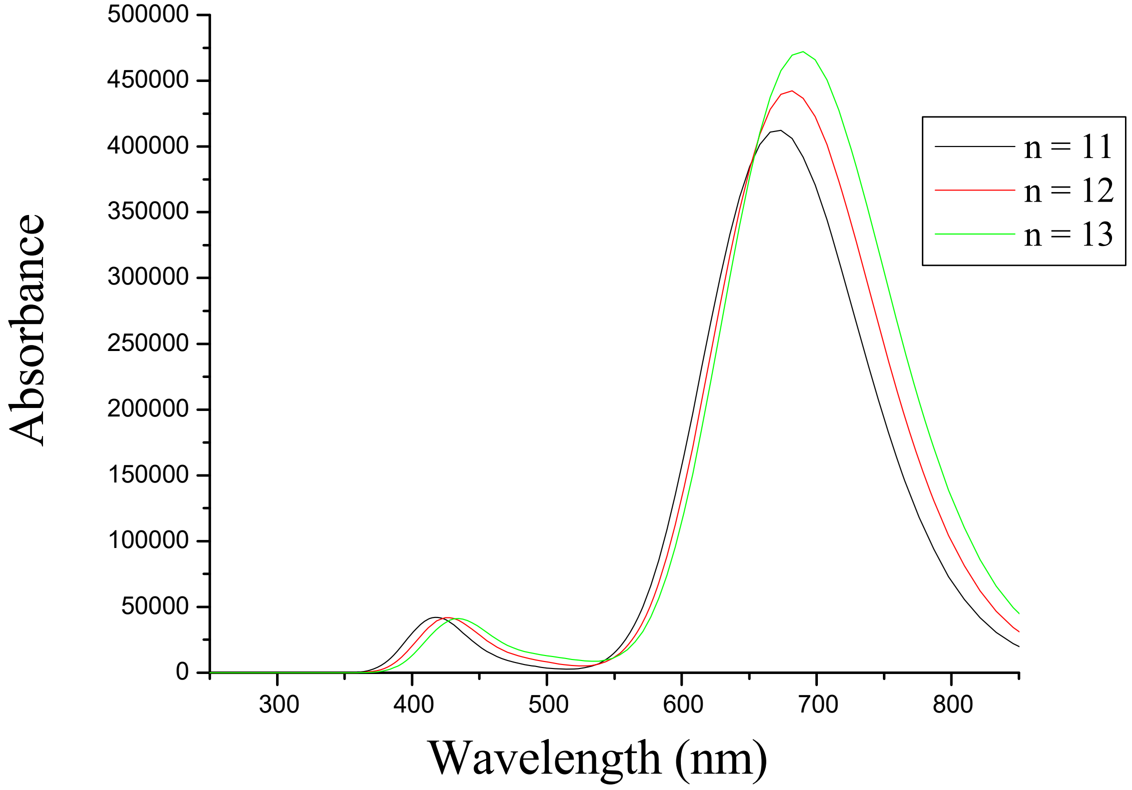

Figure S2. The absorption spectrum of dyes n =11 to n = 13 simulated by the TD-DFT-CAM-B3LYP/6-311G (d, p) level of theory for chloroform solvent.

Table S5. Electronic transition properties of dyes n =11 to n = 13 calculated at TD-DFT-CAM-B3LYP/6-311G (d, p) for chloroform solvent.

Compounds	E_{ex} (eV)	Wavelength (nm)	Oscillator Strength (*f*)	Transition	Major Contribution
**n = 11**	1.85	670.80	5.70	HOMO→LUMO	68%
**n = 12**	1.82	680.15	6.11	HOMO→LUMO	64%
**n = 13**	1.80	688.18	6.52	HOMO→LUMO	60%

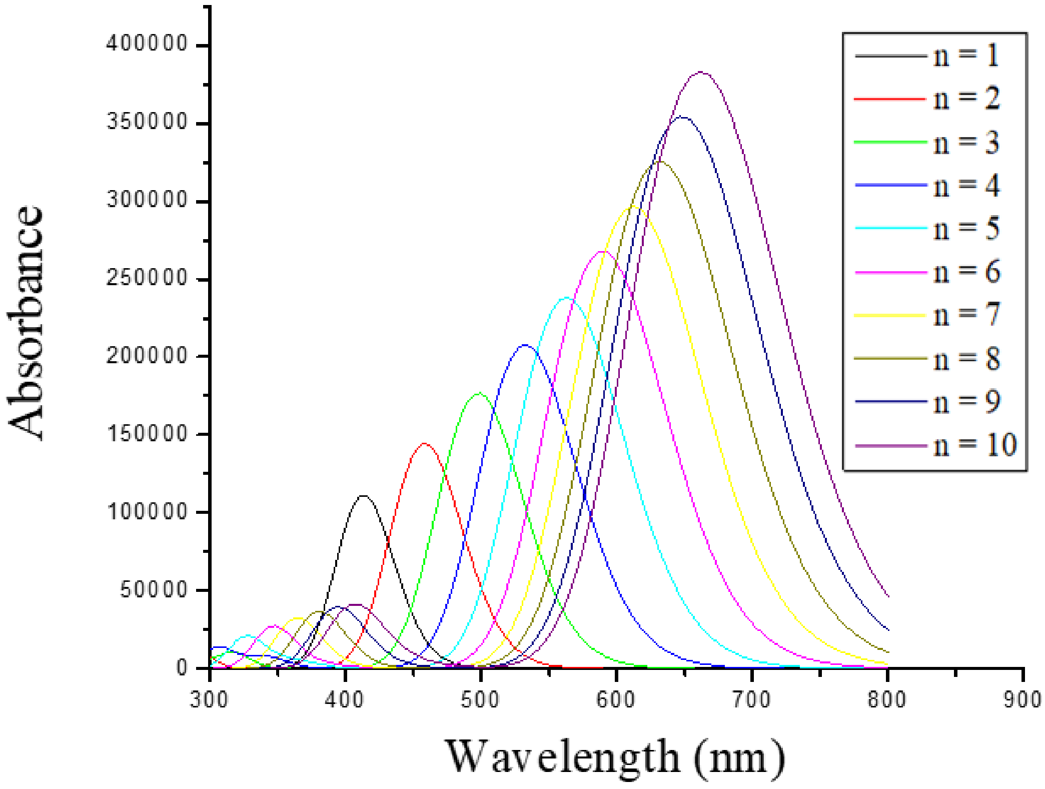

Figure S3. The absorption spectrum of dyes n =1 to n = 10 simulated by the TD-DFT-CAM-B3LYP/6-311G (d, p) level of theory for toluene solvent.

Table S6. Electronic transition properties of dyes n =1 to n = 10 calculated at TD-DFT-CAM-B3LYP/6-311G (d, p) for toluene solvent.

**Compounds**	**E_{ex} (eV)**	**Wavelength (nm)**	**Oscillator Strength (ƒ)**	**Transition**	**Major Contribution**
**n = 1**	3.00	412.647	1.53	HOMO→LUMO	96%
**n = 2**	2.71	457.675	1.99	HOMO→LUMO	94%
**n = 3**	2.49	497.169	2.44	HOMO→LUMO	92%
**n = 4**	2.33	532.00	2.87	HOMO→LUMO	90%
**n = 5**	2.20	562.486	3.28	HOMO→LUMO	88%
**n = 6**	2.11	588.91	3.696	HOMO→LUMO	85%
**n = 7**	2.026	611.693	4.097	HOMO→LUMO	82%
**n = 8**	1.97	629.09	4.496	HOMO→LUMO	79%
**n = 9**	1.926	643.538	4.894	HOMO→LUMO	75%
**n = 10**	1.883	658.354	5.293	HOMO→LUMO	71%

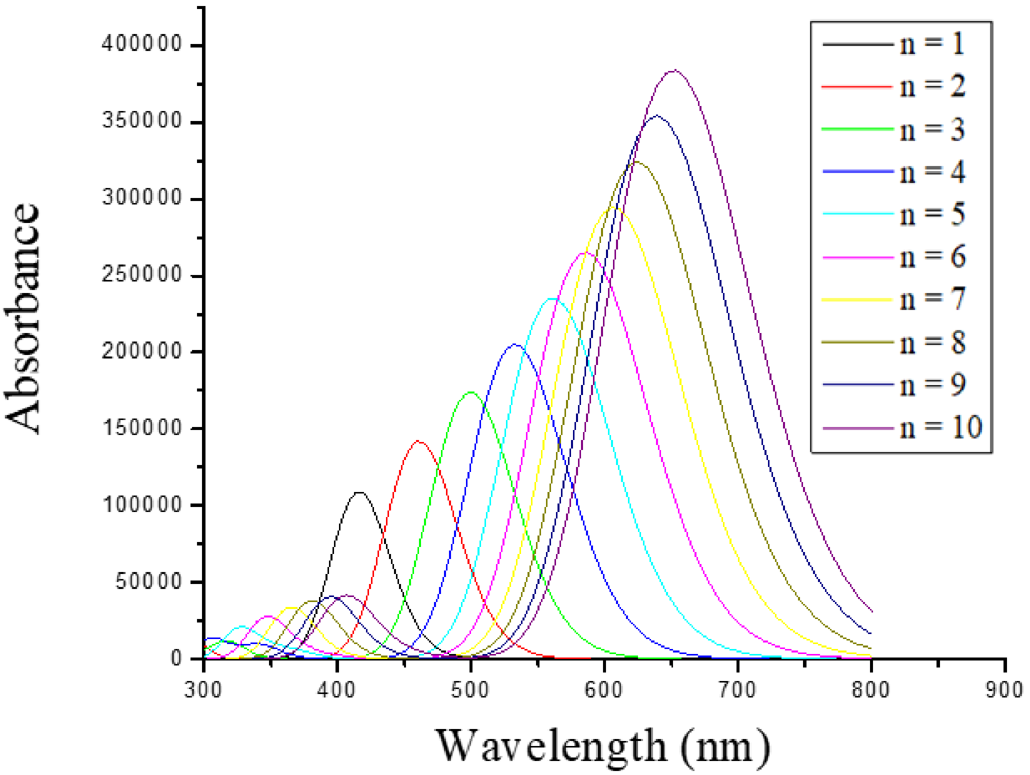

Figure S4. The absorption spectrum of dyes n =1 to n = 10 simulated by the TD-DFT-CAM-B3LYP/6-311G (d, p) level of theory for acetonitrile solvent.

Table S7. Electronic transition properties of dyes n =1 to n = 10 calculated at TD-DFT-CAM-B3LYP/6-311G (d, p) for acetonitrile solvent.

Compounds	E_{eX} (eV)	Wavelength (nm)	Oscillator Strength (ƒ)	Transition	Major Contribution
**n = 1**	3.019	410.580	1.507	HOMO→LUMO	96%
**n = 2**	2.722	455.462	1.961	HOMO→LUMO	94%
**n = 3**	2.504	495.030	2.402	HOMO→LUMO	92%
**n = 4**	2.337	530.349	2.831	HOMO→LUMO	90%
**n = 5**	2.210	560.963	3.249	HOMO→LUMO	87%
**n = 6**	2.118	585.355	3.661	HOMO→LUMO	85%
**n = 7**	2.04	606.101	4.068	HOMO→LUMO	82%
**n = 8**	1.98	623.662	4.477	HOMO→LUMO	79%
**n = 9**	1.941	638.534	4.886	HOMO→LUMO	75%
**n = 10**	1.90	651.143	5.299	HOMO→LUMO	72%

Table S8. Data describing the free energy change for dye regeneration and electron injection for dye from n = 11 to n = 13.

Compounds	E_{eX} (eV)	E dyes (eV)	E dyes* (eV)	ΔG^{inject} (eV)	ΔG^{reg} (eV)
**n = 11**	1.85	4.79	2.94	-1.06	- 0.01
**n = 12**	1.82	4.75	2.93	-1.07	- 0.05
**n =13**	1.80	4.72	2.92	-1.08	- 0.08

Table S9. The calculated light harvesting efficiency (LHE), open-circuit photovoltage (V_{OC}), and excited-state lifetime (t) for dyes n = 11 to n = 13.

Compounds	LHE	V_{OC} (eV)	τ (ns)
**n = 11**	0.999998	0.69	1.18
**n = 12**	0.999999	0.67	1.14
**n = 13**	1	0.66	1.09
